# The Bacterial Community Associated with the Amarillo Zamorano Maize (*Zea* *mays*) Landrace Silage Process

**DOI:** 10.3390/microorganisms8101503

**Published:** 2020-09-29

**Authors:** Humberto Ramírez-Vega, Ramón I. Arteaga-Garibay, Otoniel Maya-Lucas, Victor M. Gómez-Rodríguez, Ismael F. Chávez-Díaz, José M. Ruvalcaba-Gómez, Darwin Heredia-Nava, Raquel Loperena-Martínez, L. X. Zelaya-Molina

**Affiliations:** 1 Departamento de Ciencias Pecuarias y Agrícolas, Centro Universitario de Los Altos, Universidad de Guadalajara, Tepatitlán de Morelos, Jalisco 47600, Mexico; humberto.rvega@academicos.udg.mx (H.R.-V.); victor.gomez@cualtos.udg.mx (V.M.G.-R.); darwin.heredia@cualtos.udg.mx (D.H.-N.); raquel.martinez@cualtos.udg.mx (R.L.-M.); 2 Laboratorio de Recursos Genéticos Microbianos, Centro Nacional de Recursos Genéticos, Instituto Nacional de Investigación Forestales, Agrícolas y Pecuarios, Tepatitlán de Morelos, Jalisco 47600, Mexico; arteaga.ramon@inifap.gob.mx (R.I.A.-G.); chavez.fernando@inifap.gob.mx (I.F.C.-D.); 3 Departamento de Genética y Biología Molecular, CINVESTAV-Unidad Zacatenco, Ciudad de México 07360, Mexico; otto94@gmail.com; 4 Campo Experimental Altos de Jalisco, Instituto Nacional de Investigación Forestales, Agrícolas y Pecuarios, Tepatitlán de Morelos, Jalisco 47600, Mexico; ruvalcaba.josemartin@inifap.gob.mx

**Keywords:** Enterobacteriaceae, familiar-production system, heterofermentation, Lactobacillus, next-generation sequencing

## Abstract

Maize silage is used in the diet of dairy cows, with suitable results in milk yield. In this study, the composition and diversity of the bacterial communities of the silage process of Amarillo Zamorano (AZ) Mexican maize landrace with relation to the Antilope (A) commercial hybrid are described. From both types of maize, seeds were sown in experimental plots, plants harvested at the reproductive stage, chopped, and packed in laboratory micro-silos. Physicochemical parameters were evaluated, and DNA was extracted from the juice in the micro-silos. The bacterial communities were analyzed by next-generation sequencing (NGS) of seven hypervariable regions of the 16S rRNA gene. The composition of both bacterial communities was dominated by Lactobacillales and Enterobacteriales, Lactobacillales mainly in A silage and Enterobacteriales in AZ silage; as well, the core bacterial community of both silages comprises 212 operational taxonomic units (OTUs). Sugar concentration showed the highest number of significant associations with OTUs of different phyla. The structure of the bacterial communities was different in both silage fermentation processes, showing that AZ silage has a shorter fermentation process than A silage. In addition, NGS demonstrated the effect of the type of maize and local conditions on silage fermentation and contributed to potential strategies to improve the quality of AZ silage.

## 1. Introduction

Ensiling is a method used to preserve forage through a rapid decrease in pH by lactic acid fermentation, and it indeterminately maintains an acid pH under anaerobic conditions [1,2]. Thus, forage is preserved for the off-season periods when fresh forage is not available, with a minimum loss of dry matter and nutrients, while maintaining the palatability for livestock and limiting the presence of spoilage microorganisms for cattle [3]. The success of this process depends on various factors, such as crop features during the harvesting period, climatic conditions, silage establishment, intrinsic factors of the process, and silage microbiota, among others [2,4,5,6,7]. The microbial communities of silage are primarily driven from the phyllosphere of leaves and lower stems of the forage crops. however, the composition of the communities can change according to the crop and its varieties selected for the fermentation process [8].

Studies of the microbial dynamics on the silage process have provided a good understanding of the principles of ensiling and have established a key role for bacteria in the entire process [5,9]. Several bacterial species in the different silage phases have been described through studies with culture and DNA-based profiling techniques, i.e., facultative aerobic microorganisms, such as *Erwinia herbicola* and *Rahnella aquitilis* in the aerobic phase; lactic acid bacteria of *Enterococcus*, *Lactococcus*, *Lactobacillus*, *Leuconostoc*, *Paralactobacillus*, *Pediococcus*, *Streptococcus,* and *Weissella* genera, as well as different enterobacteria and clostridia in the fermentation and stabilization phases; and acetic acid and aerobic bacteria in the feeding out phase, depending on the quality of the silage [10,11,12,13,14,15,16,17,18,19,20,21,22]. Although several bacterial species have been isolated and characterized by the following method: 16S ribosomal RNA (16S rRNA) gene, massive next-generation sequencing (NGS) studies on the silages of alfalfa, grasses, small grains, maize, maize-sorghum, and soybean have provided a larger view on silage microbial community and dynamics [21,23,24,25,26,27,28,29,30,31,32]. Specifically, in maize silages, NGS studies have also shown the effects of additives or silage size on the bacterial community and silage quality of maize hybrids or corn stover [33,34,35,36,37,38,39]. However, there are no NGS studies on the composition, diversity, and succession of the bacterial communities associated with Mexican fodder maize landraces.

Maize is one of the most ensilaged crops worldwide, due to its ease of cultivation, high yield, high energy content, and good ensiling characteristics, such as low buffering capacity and high concentration of water-soluble carbohydrates [40]. Additionally, maize silage could be used alone or in association with other crops as the diet for dairy cows, e.g., grass-maize silage, has suitable results in milk yield and its protein content [41,42,43]. In the Los Altos region of Jalisco State, the main dairy basins of Mexico, which is largely under a familiar milk production system, a high proportion of crops are sown for forage production; 70–100% of livestock feed is commonly based on the stubble or silage of maize. In this region, the demand from dairy producers for livestock forage has pressed the technological development of a silage process, and the main step is the knowledge of the microbial dynamics of silages to improve the regional management of ensiling the Mexican fodder maize landraces and commercial hybrids sown in the region. Specifically, Amarillo Zamorano (AZ), which is a landrace used by 60% of the producers of the region, has a versatile combination with other concentrated products. Its production and forage quality is similar to the commercial hybrids while being adaptable to the weather conditions of the region and has a lower price for seed. 

Thus, the objective of the present study is to describe the composition and diversity of the bacterial communities associated with the silage process, and changes associated with their physicochemical factors, of the Mexican AZ maize landrace with respect to the Antilope commercial hybrid (A). Both types of maize are commonly used in the wet zone of the Los Altos milk-producing region of Jalisco. Massive sequencing of the hypervariable regions of the 16S rRNA gene at different steps of the silage process will be used. Further, the key points to improve the management and quality of AZ silage (AZS) will be established.

## 2. Materials and Methods

### 2.1. Sample Collection

Maize seeds of the Amarillo Zamorano landrace (AZ) and Antilope hybrid (A) from the Asgrow Seed Company, two different types of maize frequently ensiled in the Los Altos region, were sown in an experimental field located at Tepatitlán de Morelos Municipality (20°52′57″ N, 102°43′50″ W), Jalisco state, México. The crop of both maize types was established for the rainy season in plots of ten rows (5 × 0.8 m), in a completely randomized block design with three repetitions. Whole plants from the two central rows were harvested at one half to two-thirds milk lines maturity stages (R3-R4). Whole plants were crushed in a forage cutter RKP1800 (Raiker, Guadalajara, México) to a particle size of ~2.5 cm. Chopped forage (~1.5 kg) was packed at a density of 750 kg of green matter·m-3 into 40.0 × 10.2 cm polyvinyl chloride (PVC) pipe laboratory micro-silos, and each micro-silo was covered with a PVC cap and sealed with silicon, with a gas release valve on the top. For each plant material, a total of 30 micro-silos were incubated at ambient temperature (20–30 °C). Three micro-silos were opened, with no replacement sampling at time zero and every 10 days for a total of 90 days (T0–T9).

### 2.2. Sample Processing

At each evaluation time, the juice (50 mL) from three micro-silos was extracted using a manual press. Its pH was measured with a glass electrode pH meter (Orion 3-Star, ThermoFisher Scientific Inc., Waltham, MA, USA), temperature (ST) with a Thermometer (ACC610113, Thermo Products Inc., Germany), and sugar concentration (SC) with a refractometer (Pal-1 Atago Co. Ltd., Tokyo, Japan). To determine dry matter weight (DM), the silage samples were dried at 60 °C for 48 h in a forced-air oven (D300 Novatech, Tlaquepaque, Mexico), ground, and sieved in a 1-mm screen mill (Retsch GmbH, Haan, Germany). 

### 2.3. Statistical Analysis

The data of pH, SC, DM, and ST were subjected to variance analysis (ANOVA), and differences between the means were assessed by Tukey’s multiple comparisons. The analyses were performed using Minitab^®^ 17 statistical software (Minitab Inc., State College, PA, USA).

### 2.4. Amplification and NGS of Variable Regions of the 16S rRNA Gene

Bacterial DNA was extracted from 1 mL of a composite sample of the juice of the three micro-silos of each evaluation, according to the DNA extraction protocol in [44]. The hypervariable regions V2, V3, V4, V6–7, V8, and V9 were amplified with the Ion 16STM Metagenomics Kit, according to the manufacturer’s instructions (ThermoFisher Scientific Inc., Waltham, MA, USA) on a SelectCycler thermocycler (Select BioProduct, Life Science Research, Edison, NJ, USA).

Fifty nanograms of an equimolar pool of the amplification reactions were processed to generate the DNA libraries using the Ion Plus Fragment Library Kit™ and Ion Xpress™ Barcode Adapters 1–16 (ThermoFisher Scientific Inc.). Each step was followed by a purification with the Agencourt AMPure^®^ XP Kit, according to the manufacturer’s instructions (Beckman Coulter, Inc., Atlanta, Georgia, USA). The libraries were quantified with a high-sensitivity DNA kit in a 2100 Bioanalyzer^®^ (Agilent Technologies, Santa Clara, CA, USA). Each library was adjusted to 26 pM, and 25 µl of an equimolar pool of all samples was used for the sample emulsion PCR with the One-Touch 2 (ThermoFisher Scientific Inc.), according to the manufacturer’s instructions, and enrichment with the OneTouch Enrichment System™ (ThermoFisher Scientific Inc.). The complete sample was loaded onto a 316 v2 chip (Thermo Fisher Scientific Inc.) and sequenced on the Ion Personal Genome Machine (PGM) with HiQTM chemistry. 

### 2.5. Sequence Analysis

Sequences were analyzed using QIIME v.1.9 software [45] available in Python 2.7 for Ubuntu 17.10. The bam files obtained from the PGM were converted to fastq files, and their base quality was checked in FastQC 0.11.7 [46]. Low-quality regions and sequences (Q score < 30) were trimmed out with Trimmomatic 0.36 [47]. The operational taxonomic unit (OTU) grouping was performed in Usearch with an open-reference approach, which is a hybrid approach in which the input dataset sequences are searched against a database (closed-referencing) and the sequences which fail to cluster are given to de novo algorithm for clustering (97% correspondence) [48]. The representative phylogenetic OTUs were assigned at 80% similarity using the RDP Classifier [49]. Microbial α-diversity (Shannon and Simpson indices, Chao1 and Observed species) was determined from multiple rarefactions of the samples. β-diversity (qualitative and quantitative distances) of the samples was calculated with unweighted and weighted UniFrac. The Principal Coordinate Analysis (PCoA) generated was visualized in EMPeror [50]. The hierarchical clustering heatmap was performed using the heatmap package of R language (REF from CRAN). In addition, a multivariate association with a linear model (MaAsLin) was used to identify significant associations between microbial and phenotypic variables [51]. 

The derived variable regions of 16S rRNA gene sequences datasets were submitted to the NCBI under the Bioproject accession PRJNA531195. 

## 3. Results

### 3.1. Changes in the Chemical and Physical Characteristics of the Silages

The changes in the pH, SC (°Brix), ST, and DM of AZS and A silage (AS) through the 90-day evaluation are shown in Table 1, with statistically significant differences between the data of AZS and AS of ST and pH. Both silage processes showed notable decreases in pH at T1, SC (°Bx) at T1 and T4, a decrease in ST at T6, and increases in silage DM at T5-T6. In general, the AZS had higher values of sugar concentration (°Bx) and a more stable pH than the AS. The data for silage DM was different in both silages during the evaluated period. These differences were also statistically different (Table 1).

### 3.2. Bacterial Community Composition and Structure

A total of 2,070,194 reads were generated from the samples of both silages. After quality filtering, 85% of the total reads were kept, with 12,616–335,586 sequences of 200 nt per sample (Table 2). The bioinformatics analysis generated a total of 1,744,978 OTUs, with a minimum of 12,483 and a maximum of 330,368 OTUs per sample (Table 2). The OTUs were assigned to 243 genera, 148 families, 99 orders, 64 classes, and 25 phyla. The OTUs with an abundance of >1% corresponded to 9–24 different genera, depending on the sample. 

**Figure 1:**
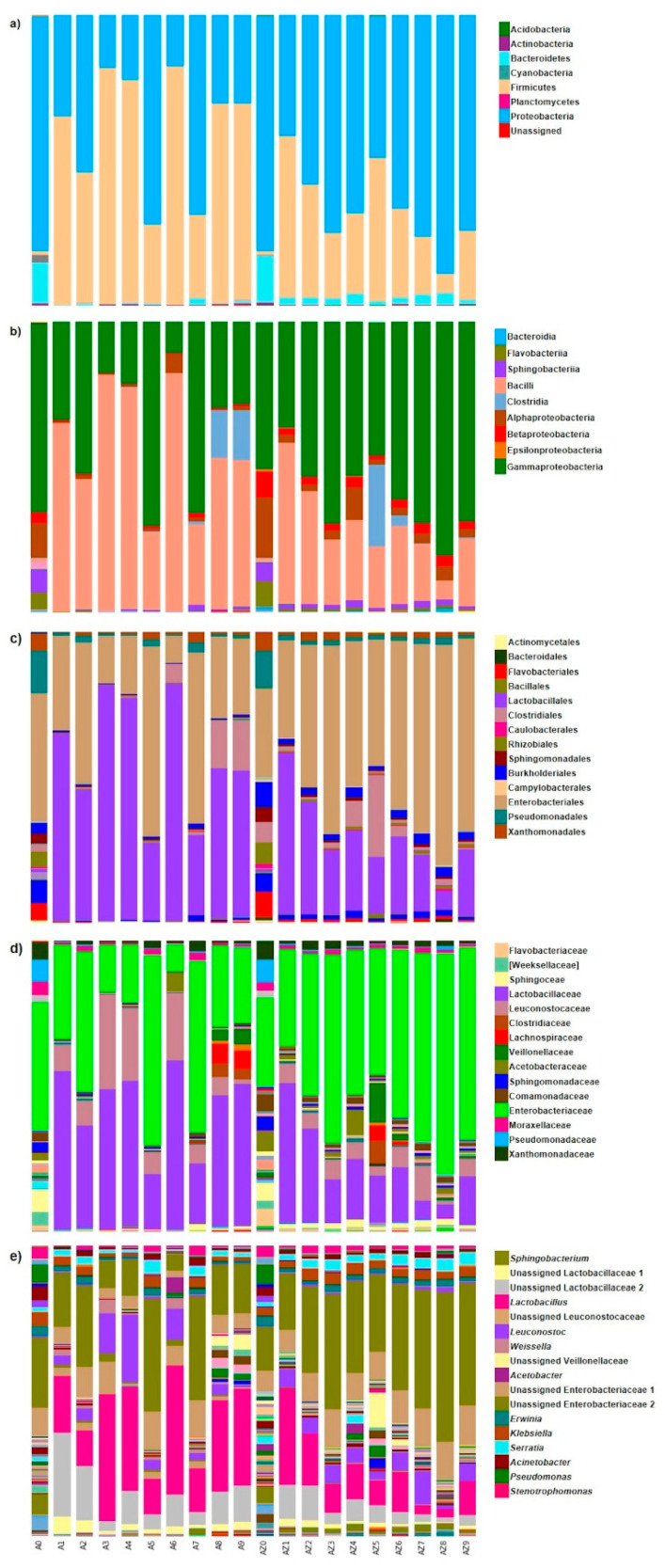
Relative abundance of dominant bacterial species of maize silage samples at the level of (**a**) phylum, (**b**) class, (**c**) order, (**d**) family, and (**e**) genus. Samples A0–A9 correspond to samples of Antilope silage, and AZ0-AZ9 to samples of Amarillo Zamorano silage, both in 10 sampling times.

The parameters of abundance and diversity indicate that the sampling depth was adequate to capture the main bacterial community of the initial plant materials and from the fermentation process of both silages (Table 3). In both fermentation processes, the richness of species and diversity indexes showed that the structure and composition of both bacterial communities have different patterns. In AZS fermentation, the species richness decreased in the first 20 days, then increased during the remaining 70 days, ending the last sample (AZ-T9) as the richest in species number. However, in AS, the non-fermented sample (T0) had the highest species richness, which diminished during the next 80 days, with a strong increase at the end of the evaluation (T9) (Table 3). In both silages, non-fermented samples (T0) had the highest values of the diversity parameters. In AZS, after the decreases of the first 20 days, the parameters also increased and remained stable during the last 70 days, but in AS, although the diversity parameters similarly diminished in the initial 20 days, they varied in the last 70 days (Table 3). The decrease in pH at T1, sugar concentration (°Bx) at T1 and T4, temperature at T6, increase in silage dry matter (T5 in AZ, T6 in A), and the change in the bacterial community could have promoted the variation of the parameters (Table 1, Figure 1).

The taxonomic composition of the silages changed through the fermentation process, and the succession of the bacterial communities was noted for the 90 days (Figure 1). Considering the OTUs with a relative abundance (RA) > 10%, at the phylum level, bacterial communities were dominated by Firmicutes (1.2–81.9% RA) and Proteobacteria (17.7–81.0% RA), Bacteroidetes were permanently existing but in a lower proportion (0.1–16.2% RA). In the initial samples (T0), the bacterial communities had a similar composition, just AS material with a larger abundance of Cyanobacteria (2.4% RA). Then, the bacterial communities were different in both silages. In AZS fermentation, Proteobacteria (41.6–89.0% RA) predominated over Firmicutes (6.4–49.2% RA) and Bacteroidetes (1.4–3.6% RA), but in AS, Firmicutes (27.0–81.4% RA) predominated over Proteobacteria (17.7–72.1% RA) and Bacteroidetes (0.1–2.3% RA) (Figure 1). This behavior was extended over the lower taxonomic levels. Consequently, at the class level in AZS, Gammaproteobacteria (36.5–80.6% RA) predominated over Bacilli (0.9–64.7% RA), Alphaproteobacteria (1.6–20.8% RA), and Betaproteobacteria (1.4–20.8% RA). In AS, Bacilli (1.2–81.8% RA) predominated over Gammaproteobacteria (10.8–70.4% RA) and Alphaproteobacteria (0.3–12.0% RA). While, at the order level, in AZS, Enterobacteriales (30.7–75.9% RA) predominated over Lactobacillales (0.7–55.5% RA), Clostridiales (<0.1–28.0% RA) and Pseudomonadales (1.2–12.9% RA). In AS, Lactobacillales (1.1–81.7% RA) predominated over Enterobacteriales (9.6–65.4% RA), Pseudomonadales (0.5–14.7% RA) and Clostridiales (<0.1–17.1% RA). Whereas, at the family level, in AZS, Enterobacteriaceae (30.7–75.9% RA) predominated over Lactobacillaceae (0.3–47.8% RA), Veillonellaceae (<0.1–13.4% RA), and Leuconostocaceae (0.1–11.9% RA). In AS, Lactobacillaceae (0.3–58.1% RA) predominated over Enterobacteriaceae (9.6–65.4% RA) and Leuconostocaceae (0.3–32.6% RA) (Figure 1). Finally, at the genus level, in AZS fermentation, an unclassified Enterobacteriaceae (15.5–51.6% RA) predominated over *Lactobacillus* (0.3–33.3% RA), a second unclassified Enterobacteriaceae (7.1–13.5% RA), an unclassified Veillonellaceae (<0.1–12.0% RA), an unclassified Lactobacillaceae (<0.1–11.9% RA) and *Leuconostoc* (0.1–11.5% RA). In AS fermentation, *Lactobacillus* (0.4–44.5% RA) predominated over an unclassified Enterobacteriaceae (5.6–39.0% RA), an unclassified Lactobacillaceae (<0.1–29.1% RA), *Leuconostoc* (0.2–23.2% RA), a second unclassified Enterobacteriaceae (2.1–13.2% RA) and an unclassified Leuconostocaceae (0.1–11.1% RA) (Figure 1). 

Additionally, in AZ, the plant material had higher RA of Flavobacteriaceae and *Dysgomonas* at T0. In all the fermentation process, AZS had higher RA of Alphaproteobacteria, Betaproteobacteria, Enterobacteriaceae, *Sphingobacterium*, *Acinetobacter*, *Pseudomonas* and *Stenotrophomonas*, and a lower RA of Lactobacillaceae and Leuconostocaceae, with regard to AS fermentation. Furthermore, there was a phase shifting in the appearance of Clostridiales in both silages. In AZS, mainly members of the Veillonellaceae family had higher RA at T5 and T6, but in AS, Lachnospiraceae had higher RA at T8 and T9 (Figure 1).

### 3.3. Cluster Analysis, Core Bacterial Community and Diversity Changes in the Ensiling Process

**Figure 2:**
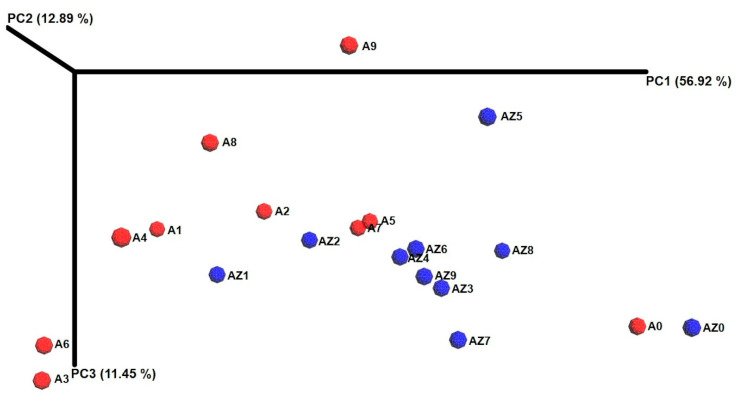
Principal coordinate analysis plot of maize silage samples, exhibiting dissimilarity of the microbial communities through the 90-days fermentation process. PC1 axis explains 56.92%, PC2 axis 12.89% and PC3 11.45% of total variance, respectively. Red dots stand for Antilope silage samples and blue dots for Amarillo Zamorano silage samples.

Compositional differences among the bacterial communities of both silages are shown in the weighted principal coordinate analysis (PCA) UniFrac plot (Figure 2). Distinct clusters were identified in relation to the silage fermentation time grouping A0 and AZ0 as the starting clusters, and A1, AZ1, A2, and AZ2 as the early ensiling-cluster. The subsequent AZS samples corresponded to a middle-later ensiling-cluster, except AZ5, likely a crucial point in AZS fermentation. However, between A3–A9 silage samples, there was no clear clustering through the silage fermentation process. Rather, the small clusters (A1/A4, A3/A6, A5/A7) showed abrupt changes in the most abundant and shared OTUs of the bacterial community of these samples during the fermentation (Figure 2).

**Figure 3:**
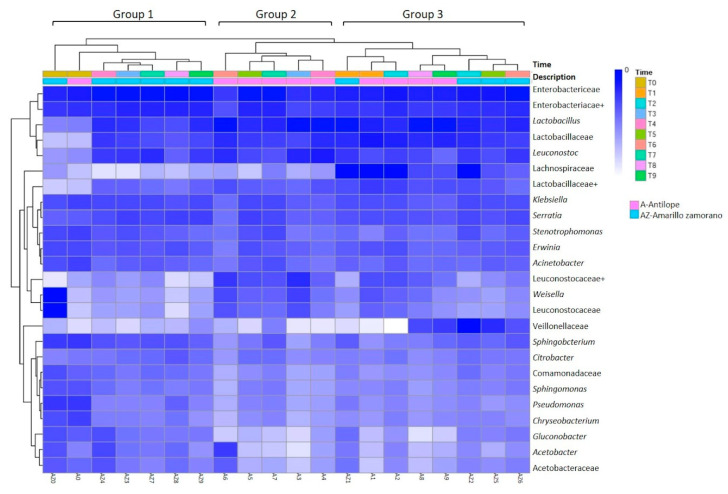
Heatmap plot illustrating the top 25 bacterial operational taxonomic units (OTUs) (*Y*-axis) of the silage samples (*X*-axis). The relative values for bacterial OTUs are depicted by the color intensity with the legend indicated at the bottom of the figure. Clusters based on the distance of the samples along the *X*-axis and the bacterial OTUs along the *Y*-axis are indicated in the upper and left of the figure, respectively.

The core bacterial community comprised 212 OTUs that were present in all the samples of both ensiling processes, including the non-fermented and all the fermented samples. They corresponded to just 0.012% of the total OTUs determined in this study. At the genus level, the core bacterial community grouped into 27 OTUs: Flavobacterium, Corynebacterium, *Sphingobacterium*, *Lactobacillus*, *Leuconostoc*, *Lactococcus*, *Ochrobactrum*, *Devosia*, *Agrobacterium*, *Acetobacter*, *Gluconobacter*, *Sphingomonas*, *Comamonas*, *Delftia*, *Limnohabitans*, *Polaromonas*, *Citrobacter*, *Enterobacter*, *Erwinia*, *Gluconacetobacter*, *Klebsiella*, *Morganella*, *Plesiomonas*, *Serratia*, *Acinetobacter*, *Pseudomonas*, *Stenotrophomonas*, and eight unclassified genera of Lactobacillaceae, Acetobacteraceae, Brucellaceae, Sphingomonadaceae, Comamonadaceae, Enterobacteriaceae, Pseudomonadaceae, and Xanthomonadaceae families. Moreover, there was an OTU from an unclassified Pseudomonadales and another from Streptophyta (by the chloroplasts of plants). Members of the bacterial community with major RA could be observed in the Heatmap analysis (Figure 3), together with the other five OTUs with high RA: Weisella, and unclassified Veillonellaceae, an unclassified Lachnospiraceae, and two unclassified Leuconostocaceae. 

The microbial communities associated with the AZS and AS fermentation process also shared some bacterial OTUs. These OTUs varied in abundance during the whole processes (T1–T9), as can be observed in the heatmap analysis (Figure 3). The heatmap analysis also suggests that the OTUs were grouped in three main clusters: the first one with OTUs with high abundance during the fermentation process (Enterobacteriaceae, Enterobacteriaceae+, *Lactobacillus*, Lactobacillaceae, *Leuconostoc*), a second cluster with OTUs of lower abundance (Lactobacillaceae, *Klebsiella*, *Serratia*, *Stenotrophomonas*, *Erwinia*, *Acinetobacter*), and the third cluster of OTUs with the lowest abundance (*Sphingobacterium*, *Citrobacter*, an unclassified Comamonadaceae, *Sphingomonas*, *Pseudomonas*, *Chryseobacterium*, *Gluconobacter*, *Acetobacter*, and an unclassified Acetobacteraceae). As well, there are key OTUs at specific time points: an undefined Lachnospiraceae at A1, A2, AZ1, and AZ2; an unclassified Veillonellaceae at AZ2, AZ5, A8 and A9; and unclassified Leuconostocaceae and *Weisella* at AZ0, A6 and A3; and *Acetobacter* at A6. Based on the abundance of these OTUs, the silage samples were assembled into three groups, differentiating the middle AS from the other samples (Group 2: A3–A7). Group 1 had samples consisting of middle and later AZS (AZ3, AZ4, AZ7, AZ8, AZ9), and Group 3 had early and middle AZS (AZ1, AZ2, AZ5, AZ6) and early and later AS (A1, A2, A8, A9). It presents that AZS had a shorter fermentation process than AS, signifying that the bacterial succession was also shorter in AZS due to the change in the main OTUs in the bacterial community (Figure 3). 

### 3.4. Significant Association between Microbiota and Chemical and Physical Characteristics of Silage

**Figure 4:**
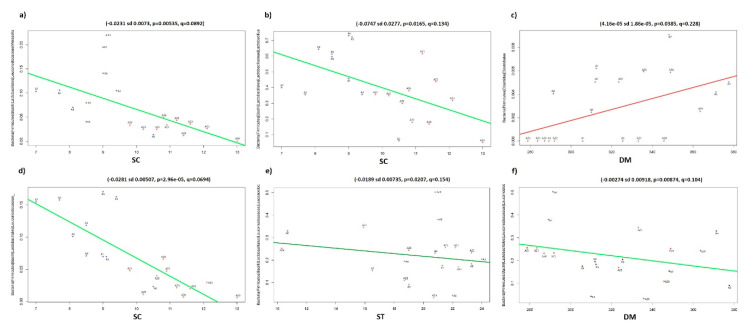
Multivariate linear associations of silages metadata and bacterial relative abundance. Scatter plots show the significant associations of sugar concentration (SC), silage temperature (ST), or dry matter weight of 1 kg of silage (DM) with different Firmicutes: (**a**) *Weissella*, (**b**) *Lactobacillus* (**c**) Clostridiales, (**d**) Leuconostocaceae, (**e**) and (**f**) *Leuconostoc*; y-axes show the relative abundance of silage bacterial community and x-axes show the silage metadata.

In the multivariate association with linear models (MaAsLin) analysis between maize silages metadata and relative abundance of silages microbiota, the variable of sugar concentration (SC) showed the highest number of significant associations with OTUs of different phyla. A negative association was exhibited between SC and 65 OTUs of Proteobacteria, mainly alphaproteobacteria, one of Firmicutes, eight of Bacteroidetes, five of Acidobacteria, and four of Actinobacteria; and a positive association with eight OTUs of Firmicutes, mainly genera of Lactobacillales. OTUs of Proteobacteria were also the most negatively associated with pH (*n* = 8), dry matter weight of 1 kg of silage (DM, *n* = 9), and silage temperature (ST, *n* = 6). In addition, some of the Firmicutes OTUs with high relative abundance were associated with SC, pH, and ST (Figure 4). 

## 4. Discussion

Data from NGS studies on different crop silages have shown a wide view of the silage microbial composition and dynamics [26,29,30]. Thus, in the present study, the bacterial community associated with the maize silage process of a forage Mexican landrace and a commercial hybrid, both commonly used in the Los Altos region, Jalisco, were surveyed by a massive high-throughput sequencing approach.

The high bacterial composition and diversity of AZS and AS during the entire fermentation process (Table 3) contrasts with the previously reported lower values for maize and other crops silages, as the result of a low pH and 80–95% of relative abundance of the *Lactobacillus* genus [23,27,28,29,30,33], but similar to the high values of diversity obtained from the silages of alfalfa, maize from Iran [26,33], and other fermentative processes. Although the AS and AZS had a low pH in all evaluations, the lower relative abundance of *Lactobacillus* in AS and AZS (0.3–44.5%) reflects that other members of the microbial community were involved in this process, likely as a result of the type of maize, stage of maturity at harvest, local conditions during crop growth or climate [52].

The process of silage fermentation is based on the production of lactic acid by homofermenters and heterofermenters that inhibit the growth of spoilage microorganisms and the activity of plant enzymes [28,53,54]. Culture and culture-independent techniques, such as DGGE, T-RFLP, and NGS have described members of Lactobacillales (*Lactobacillus*, *Lactococcus*, *Pediococcus,* and/or *Weissella*) as the main heterofermenters (70 to 95% of the bacterial population) in the silage process. Although the initial population of Lactobacillales is extremely small in pre-ensiled crops by its low abundance as epiphytic and endophytic populations in forages, these microbes become predominant in the silage bacterial community by the intense selection process of ensiling, particularly the depletion of oxygen and inhibition of the metabolism of other microorganisms by acidification. Over time, lactic acid bacteria (LAB) tend to decrease during storage, and other bacterial species, including Proteobacteria (Xanthomonadales, Sphingomonadales, Enterobacteriales) and Actinomycetales, as the remaining members of the microbiome, can increase in abundance within the silage [23,28,30,55,56]. The bacterial communities’ composition of AS and AZS varied throughout the 90-day evaluation and by the type of maize, reinforcing the idea of the effect of type of maize used for the silages and the bacterial communities’ succession as a result of the metabolic processes of the silage environment. In both silages, a pH < 4.0 was constant, almost during the entire fermentation process, suggesting the production of lactic acid by the hetero-fermentation of the bacterial community. However, the pH increase in AS-T7, AS-T8, and AS-T9 could result from the production of acetic acid or butyric acid, possibly related to the increase in Enterobacteriaceae, Clostridiaceae, or Veillonellaceae OTUs. Some species of these families can be proteolytic and produce ammonia, amines, and carbon dioxide. Other species can ferment carbohydrates or lactic acid and produce butyric acid, acetic acid, hydrogen, and carbon dioxide, products that reduce the silage intake by livestock [20,57].

Overall, both silages had a bacterial community succession similar to those previously reported. Mainly in AS, members of *Lactobacillus* genera were dominant in the fermentation process, with a variable RA of other Firmicutes, Proteobacteria, and Bacteroidetes. But in AZS, Enterobacteriaceae OTUs had higher relative abundance during the fermentation process than all the Firmicutes and Bacteroidetes, as was reported in whole-plant maize silage from China [36]. Although Enterobacteriaceae species were the main competitors of LAB for the sugars in the silage, strains of *Citrobacter*, *Morganella*, *Plesiomonas*, *Serratia*, *Enterobacter*, *Erwinia*, *Klebsiella*, *Morganella*, and also from the undefined enterobacteria genera, could play key roles in different stages of the silage process. This bacterial community and its principal fermentation products [20,58] must be related to the acidified environment or at least maintained under this condition. Furthermore, it was able to cushion slightly more pH and likely other silage conditions. As previously reported in grass silage, the aerobic stability was found to be increased by high numbers of enterobacteria, and also by the fermentation end products of clostridia [59,60,61]. Additionally, in AZ, the high RA of *Weisella* and a Leuconostocaceae OTU at T0, as part of the phyllosphere of AZ, probably supported the beginning of the fermentation process.

About the other members of the core bacterial community or the OTUs with high RA, almost all have been reported previously as members of the community of maize silage, other crop silages, or food fermentation processes. *Flavobacterium* has been reported in corn silage and can degrade the cellulose fiber of maize [36,62]. *Chryseobacterium* has been detected in tropical grass–legumes silages producing high contents of acetic acid and butyric acid and has also been associated with a different process of maize fermentation [63,64]. *Sphingobacterium* has been identified in pre-ensiled maize and could be involved in ethanol production [30].

*Ochrobactrum* has been found mainly in fresh maize-plant material [30,34,38] with cellulolytic activity for degrading maize material [65]. Meanwhile, *Devosia* has been reported in the pre-ensiled of Italian ryegrass, whole corn crop, and pre-ensiled and silage of alfalfa. There is no information related to its participation in silages [30], but strains of this genus have been characterized for their capacity to detoxify deoxynivalenol [66], a toxin existing in the maize crop. *Agrobacterium* was also found in pre-ensiled and silages of Italian ryegrass, whole corn crop, and alfalfa, and there is even less information about its effect on silages [30]. 

*Gluconobacter*, *Gluconacetobacter,* and *Acetobacter* are acetic acid producers [67] and can contribute to pH decline at the early stages of silages, as well as *Acetobacter* oxidize ethanol to acetic acid [68]. *Sphingomonas* and *Pseudomonas* were found in alfalfa silage and may contribute to protein preservation [29,30]. *Comamonas* was found in corn straw and sugarcane silages and from spoiled cereal silage [27,69]. *Delftia*, *Limnohabitans,* and *Polaromonas* are genera with no previous reports on silages. Strains of *Sphingomonas* and from the group *Delftia*/*Comamonas* are involved in the degradation of Fumonisin B1, another mycotoxin produced by *Fusarium* species, which is very common in maize-based aliments [70,71]. *Limnohabitans* and *Polaromonas* species are related to marine and fresh-water [72,73,74]. *Stenotrophomonas* exists in the whole fermentation process of corn stover silage in China and could be involved in the degradation of the lignocellulosic biomass [34,75]. 

There are few reports of *Acinetobacter* as part of the silage microbiota, inferring that the strains use acetate as a carbon source, and may explain the DM losses of silages [29,76,77]. *Leuconostoc*, *Lactococcus* are known species of the fermentation phase of silage. Veillonellaceae and Lachnospiraceae OTUs are mainly associated with the ruminal microbiome of cows with corn silage-based diets. In addition, some strains of Veillonellaceae can ferment lactate to produce CO_2_, H_2_, and various lower volatile fatty acids containing 2–6 C atoms [56]. Lachnospiraceae is a fiber-degrader family, with multiple carbohydrate–hydrolyze enzymes [78]. 

PCA, heatmap analysis, and MaAsLin analysis also support the effect of type of maize and local conditions on AZ and A silage fermentation, along with the selective pressures and metabolic characteristics of a silage fermentation process. As well, the structure and composition of the AZS bacterial community suggest a shorter fermentation process in this plant material, a key point for local framers to improve the quality efficiency and management of their silages.

However, more research is needed to elucidate the role of all the bacterial genera anteriorly mentioned, as an analysis of the composition of the bacterial communities in the first 2 weeks, and culture-based studies, particularly considering that almost all the studies have been realized in maize hybrids and not in a Mexican maize fodder-landrace. The metagenome research of AZS demonstrates a new approach to study the bacterial composition involved in the fermentation process of Mexican maize fodder landraces, suggesting potential strategies to improve the silage quality of Amarillo Zamorano, permitting the cost reduction in feeding livestock and enhancing the sustainability of the familiar milk production system of the Los Altos region.

## Figures and Tables

**Table 1:** Chemical and physical characteristics of Amarillo Zamorano (AZ) and A (A) silages.

SD	SC (°Bx)		pH		ST (°C)		DM (g)	
	AZ ^a^	A ^b^	AZ ^a^	A ^b^	AZ ^a^	A ^a^	AZ ^a^	A ^a^
T0	13.0 ^A^	10.5 ^A^	6.5 ^A^	6.4 ^A^	20.7 ^ABC^	22.0 ^AB^	335.7 ^AB^	310.0 ^BC^
T1	11.2 ^AB^	9.0 ^AB^	3.7 ^B^	3.7 ^C^	22.2 ^AB^	21.3 ^ABC^	283.5 ^CD^	305.7 ^C^
T2	11.6 ^AB^	9.4 ^AB^	3.6 ^B^	3.7 ^C^	23.3 ^A^	24.0 ^A^	291.8 ^BCD^	325.4 ^ABC^
T3	12.1 ^AB^	9.1 ^AB^	3.8 ^B^	3.8 ^C^	21.5 ^A^	21.0 ^A^	278.7 ^D^	289.1 ^C^
T4	9.8 ^B^	8.1 ^BC^	3.9 ^B^	3.8 ^C^	20.8 ^ABC^	20.8 ^ABC^	287.0 ^BCD^	291.3 ^C^
T5	10.6 ^AB^	7.7 ^BC^	3.7 ^B^	3.9 ^BC^	22.4 ^AB^	23.3 ^A^	323.5 ^ABCD^	312.4 ^BC^
T6	10.8 ^AB^	9.0 ^BC^	3.8 ^B^	3.9 ^BC^	10.3 ^E^	10.7 ^E^	348.9 ^A^	371.3 ^AB^
T7	10.9 ^AB^	7.0 ^C^	3.9 ^B^	4.3 ^B^	15.9 ^D^	16.5 ^D^	333.1 ^ABC^	348.2 ^ABC^
T8	11.4 ^AB^	8.5 ^BC^	3.8 ^B^	4.0 ^BC^	18.7 ^CD^	18.7 ^CD^	345.7 ^A^	311.9 ^BC^
T9	10.2 ^B^	8.5 ^BC^	3.9 ^B^	4.1 ^BC^	19.0 ^BCD^	19.0 ^BC^	363.5 ^A^	377.6 ^A^

SC = sugar concentration, ST = silage temperature, DM = dry matter weight of 1 kg of silage, SD = sampling date, AZ= Amarillo Zamorano maize landrace, A = Antilope maize hybrid. a, b = groups of Tukey’s test with α = 0.05 between AZ and A silages of pH, SC, ST, and DM data, A, B, C, D, E, AB, BC, CD, ABC, BCD, ABCD = groups of Tukey’s test significantly different with α = 0.05, through the 10 evaluations of each variable of AZ and A silages.

**Table 2:** Number of raw 16S rDNA gene amplicon reads and bacterial phylotypes defined to 90% identity of Amarillo Zamorano (AZ) and A (A) silages.

Samples	Total Reads		Filtered Reads		OTUs No.	
	AZ	A	AZ	A	AZ	A
T0	34,027	402,085	28,977	335,586	28,381	330,368
T1	45,868	82,539	39,331	71,318	38,868	70,636
T2	14,899	134,564	12,616	112,439	12,483	111,426
T3	84,956	99,670	71,264	91,625	70,379	90,737
T4	55,867	67,526	46,695	61,511	46,148	60,859
T5	53,396	92,516	45,058	78,662	44,312	77,880
T6	69,129	125,646	58,648	114,966	58,044	113,939
T7	162,971	42,917	138,126	36,763	136,555	36,380
T8	120,059	29,305	101,028	25,460	99,779	25,153
T9	188,816	163,438	158,288	140,544	154,970	137,681

No. OTUs = number of operational taxonomic units.

**Table 3:** Microbial richness and diversity of 16S rDNA libraries based on 90% identity of operational taxonomic units (OTUs) from Amarillo Zamorano and Antilope silages samples.

Samples	Observed Species		Chao1 Index		Shannon Index		Simpson Index	
	AZ	A	AZ	A	AZ	A	AZ	A
T0	2302.8	3295.9	4018.164	5703.145	8.658	8.137	0.994	0.990
T1	1800.7	1969.7	3727.649	3606.644	6.255	5.790	0.947	0.947
T2	756.8	2194.3	1701.906	4023.674	6.608	6.613	0.973	0.972
T3	2456.7	1755.0	4697.109	3217.592	7.188	5.186	0.981	0.905
T4	2185.5	1882.2	3953.208	3518.960	7.279	5.690	0.981	0.944
T5	2047.2	2181.8	3606.409	3870.192	7.324	6.829	0.981	0.977
T6	2324.9	1767.3	4511.745	3375.423	7.100	5.364	0.979	0.919
T7	2745.7	1643.2	5016.328	3049.496	7.199	6.925	0.979	0.977
T8	2699.0	1112.7	4592.017	2134.499	7.105	6.650	0.974	0.966
T9	3133.0	2798.6	5833.050	5277.361	7.198	6.732	0.979	0.971

AZ = Amarillo Zamorano, A = Antilope.
